# No standard modifiable cardiovascular risk factors in acute myocardial infarction: prevalence, pathophysiology, and prognosis

**DOI:** 10.1007/s12928-024-01022-4

**Published:** 2024-06-17

**Authors:** Yuichi Saito, Kenichi Tsujita, Yoshio Kobayashi

**Affiliations:** 1 Department of Cardiovascular Medicine, Chiba University Graduate School of Medicine, 1-8-1 Inohana, Chuo-ku, Chiba, Chiba 260-8677 Japan; 2 Department of Cardiovascular Medicine, Graduate School of Medical Sciences, Kumamoto University, Kumamoto, Japan

**Keywords:** Hypertension, Diabetes, Dyslipidemia, Smoking, Myocardial infarction

## Abstract

Standard modifiable cardiovascular risk factors (SMuRFs), such as hypertension, diabetes, dyslipidemia, and current smoking, are associated with the development of atherosclerotic cardiovascular diseases including acute myocardial infarction (MI). Thus, therapeutic approaches against SMuRFs are important as primary and secondary prevention of cardiovascular diseases. In patients with acute MI, however, the prognosis is counterintuitively poor when SMuRFs are lacking. The growing evidence has explored the prevalence, pathophysiology, and prognosis of SMuRF-less patients in acute MI and suggested the potential underlying mechanisms. This review article summarizes the clinical evidence and relevance of the lack of SMuRFs in acute MI.

## Introduction

Cardiovascular diseases, represented by acute myocardial infarction (MI), are the most common non-communicable disorders globally and account for approximately one-third of all deaths worldwide [1–3]. Standard modifiable cardiovascular risk factors (SMuRFs), such as hypertension, diabetes, dyslipidemia, and current smoking, are associated with the development of cardiovascular diseases in a significant magnitude [4, 5]. A recent pivotal study with pooled individual-level data of more than a million participants across geographic regions demonstrated that nearly 60% of cases of incident cardiovascular disease are attributable to only five SMuRFs (the four factors above plus obesity) [5]. The identification and targeted treatment of SMuRFs can reduce the burden of cardiovascular events as primary and secondary prevention [6–8]. When focusing on patients who developed acute MI, however, it has been recently acknowledged that the prognosis is counterintuitively poor in those having none of the SMuRFs as compared to patients with at least one SMuRF (Table 1) [9–32]. The prevalence of SMuRF-less patients varies widely in the previous reports with possible geographic differences. In addition, the poor clinical outcomes in SMuRF-less patients may be time-dependent after acute MI with presumable underlying mechanisms. The current understanding, evidence, and guideline recommendations for ischemic heart disease may not be applicable to patients with no SMuRFs [33–72]. This narrative review article provides a practical overview of the current evidence of the lack of SMuRFs in acute MI.

**Table 1:** Key studies investigating the impact of no SMuRFs in ACS

References	Publication year	Study period	Region/country	Sample size	Clinical presentation	Study population	PCI (%)	No SMuRFs (%)	In-hospital mortality (vs. ≥ 1 SMuRFs)
#9	2007	2001–2004	United States	74,220	NSTEMI	All-comers	39	10	7% vs. 5%
#10	2014	1985–2008	The Netherlands	14,434	Acute MI	No prior CAD	53	31	7% vs. 5%^a^
#11	2015	1999–2008	Canada	13,686	ACS	All-comers	34	14	5% vs. 3%
#12	2017	2006–2014	Australia	695	STEMI	No prior CVD	NA	25	1% vs. 1%
#13	2018	2000–2016	United States	1475	Acute MI	≤ 50 years	NA	17	NA
#14	2019	1999–2017	Australia	3081	STEMI	No prior CVD	45	19	6% vs. 4%
#15	2021	2005–2018	Sweden	62,048	STEMI	No prior CAD	71	15	10% vs. 7%
#16	2022	2005-2013^b^	Japan	8312	STEMI	PCI/CABG	98	4	15% vs. 5%^c^
#17	2022	2002-2011^d^	Global	2862	STEMI	PCI	100	18	1% vs. 3%^c^
#18	2022	2008–2018	Singapore	22,160	Acute MI	No prior CAD	100	5	HR 1.45 (1.11–1.90)^e^
#19	2022	2010–2017	United Kingdom	176,083	NSTEMI	No prior CAD	47	23	5% vs. 5%
#20	2022	2012–2020	Japan	1093	Acute MI	PCI	100	6	23% vs. 8%
#21	2022	2005–2018	Sweden	99,718	NSTEMI	No prior CAD	52	11	4% vs. 3%
#22	2022	2011–2017	Singapore	5400	Acute MI	No prior CAD	73	9	12% vs. 6%^c^
#23	2022	2013–2014	China	16,228	STEMI	No prior CAD	64	12	8% vs. 6%
#24	2023	2019–2020	Japan	115,437	Acute MI	PCI	100	2	18% vs. 5%
#25	2023	2010–2020	Australia	597	STEMI	Age 18–45 years	99	8	10% vs. 2%^c^
#26	2023	2013–2021	Pakistan	15,051	ACS	No prior CVD	47	15	4% vs. 4%
#27	2023	2010–2014	Global	23,489	ACS	In-hospital survivor	72	20	NA
#28	2023	2015–2019	Iran	7847	ACS	PCI	100	11	2% vs. 2%
#29	2023	2010–2014	United States	474,234	STEMI	PCI	100	11	8% vs. 3%
#30	2023	2018–2019	India	2379	STEMI	No prior CAD	15	25	11% vs. 11%
#31	2023	2009–2023	Japan	7775	ACS	PCI	100	7	10% vs. 4%
#32	2024	2014–2019	China	10,775	STEMI	≥ 75 years	66	15	5% vs. 5%

SMuRFs include hypertension, diabetes, dyslipidemia (or hypercholesteremia), and current smoking

*ACS* acute coronary syndrome, *CABG* coronary artery bypass grafting, *CAD* coronary artery disease, *CVD* cardiovascular disease, *HR* hazard ratio, *MI* myocardial infarction, *NA* not applicable, *NSTEMI* non-ST-segment elevation myocardial infarction, *PCI* percutaneous coronary intervention, *SMuRF* standard modifiable cardiovascular risk factor, *STEMI* ST-segment elevation myocardial infarction

^a^Age-adjusted 30-day mortality

^b^Two separate study periods (2005–2007 and 2011–2013) were included

^c^Mortality at 30 days

^d^Pooled data from 10 randomized-controlled trials

^e^HR with 95% confidence intervals

### Prevalence of no SMuRFs in acute MI

**Fig. 1:**
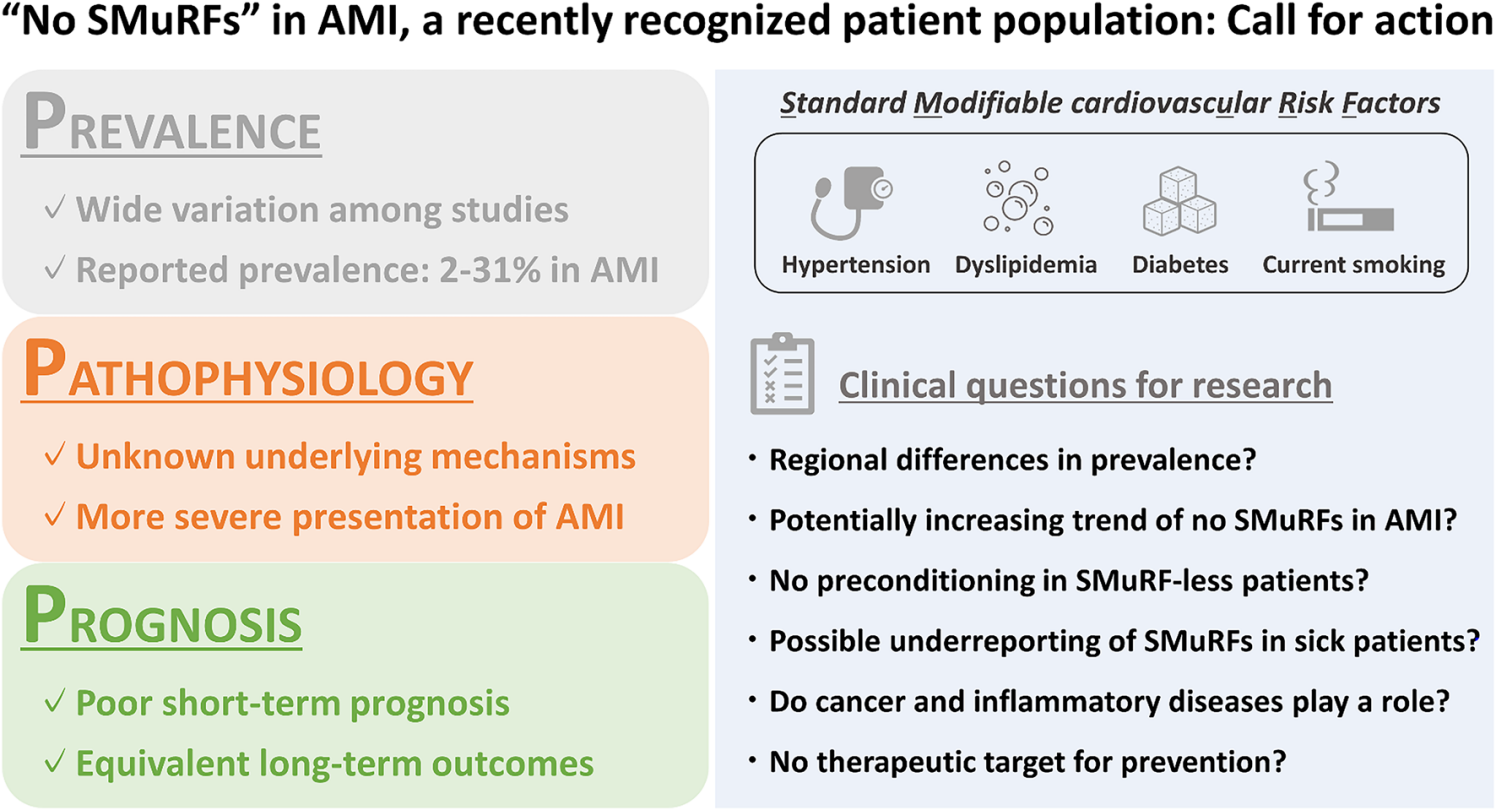
Prevalence, pathophysiology, and prognosis of patients with acute myocardial infarction and no standard modifiable cardiovascular risk factors. *AMI* acute myocardial infarction, *SMuRF* standard modifiable cardiovascular risk factor

In the field of atherosclerotic cardiovascular disease, SMuRFs play significant roles. Treatment strategies against SMuRFs have been established as effective primary and secondary prevention for over 50 years [73], along with numerous investigations for the risk factors [74–84]. In daily clinical practice, however, we as a clinician often encounter patients who have none of the SMuRFs in a setting of acute MI. A meta-analysis including 1,285,722 patients with acute coronary syndrome (ACS) from 15 studies across regions reported that the prevalence of being SMuRF-less was overall 11.6% [85]. In key previous studies in this literature as shown in Table 1, the prevalence varied widely, ranging from 2 to 31%, which may, at least partially, depend on different definitions of SMuRFs [86]. For instance, some studies employed dyslipidemia as a part of SMuRFs [10, 24], while hypercholesteremia was defined as one of the SMuRFs in others [15, 32]. In addition, “current” smoking was defined as a history of smoking in the past year [24, 32], within 1 month [15], and others. In addition, regional or racial differences may exist in the prevalence of no SMuRFs. As shown in Table 1, the prevalence in studies from East Asia (Japan, China, and Singapore) ranged from 2 to 15%, while from 8 to 31% in Western countries (United States, Canada, Australia, United Kingdom, Sweden, and The Netherlands). Indeed, in the above-mentioned meta-analysis, the proportion of SMuRF-less ACS patients in each geographical region was reported to be lowest in Asia (7.5%), followed by Europe (10.1%), North America (11.7%), and Australia (21.3%) [85]. Nevertheless, it remains unclear whether the regional variation is derived from racial and ethnicity-based differences or is associated with a healthcare system (e.g., annual health checks) and the quality of data collection. Another important point for debate is a potentially increasing trend of the proportion of no SMuRFs in patients with acute MI during recent decades. Early Australian studies indicated an increasing proportion of ST-segment elevation MI (STEMI) patients with no SMuRFs until the mid-2010s [12, 14], while subsequent larger registries in Sweden and the United Kingdom showed no significant trend in the proportion in STEMI and non-ST-segment elevation MI (NSTEMI) [15, 19, 21]. A multi-ethnic registry in Singapore reported that the increasing trend of the proportion of SMuRF-less patients was found only in STEMI but not in NSTEMI [18]. The temporal trend of patients with acute MI and no SMuRFs may be ethnicity- and geography-specific but deserves further investigation (Fig. 1).

### Pathophysiology of SMuRFs-less patients

The pathophysiology of patients with acute MI and no SMuRFs has also been investigated. Although underlying mechanisms of acute MI in patients who do not have any SMuRFs remain to be established, non-standard risks should be acknowledged, such as cancer, inflammatory conditions, nutrition, thrombotic factors, environmental and occupational exposures, mental, sleep and oral health, physical activity, social factors, and genetics [86–105]. It is conceivable that SMuRF-less acute MI patients are heterogeneous populations. Therefore, comprehensive screening for such non-standard risk factors is clinically relevant. Indeed, we previously reported that up to one-third of patients with acute MI and no SMuRFs had active cancer and chronic systemic inflammatory diseases (CSIDs) (e.g., rheumatoid arthritis and inflammatory bowel disease) [20, 106], both of which are associated with an increased risk of thrombotic/ischemic events [107, 108]. Importantly, in a setting of acute MI, SMuRF-less patients are likely to be presented with more severe manifestations, including STEMI rather than NSTEMI, left main coronary disease, cardiogenic shock, and cardiac arrest. The proportion of being SMuRF-less is reportedly higher in patient with STEMI than in those with NSTEMI in the previous studies [10, 11, 16, 18, 22, 24, 26, 27]. A Japanese large-scale registry showed that the proportion was even lower in patients with chronic coronary disease (2.5%) than in those with STEMI (4.4%) and NSTEMI (3.2%) [16]. From an angiographic perspective, MI is more likely to be attributable to the left main disease in SMuRF-less patients than in those with at least one SMuRF, particularly in NSTEMI [16, 24]. On the other hand, multivessel coronary artery disease was less frequently observed in acute MI patients with no SMuRFs [109]. In addition, a serial intravascular imaging study showed that SMuRF-less patients with established coronary disease had less atheroma and calcification volumes at baseline and similar rates of coronary plaque progression as compared to those with risk factors [110]. Another mechanistic study with pooled data from 10 randomized percutaneous coronary intervention (PCI) trials showed that SMuRF-less patients more frequently had poor pre-PCI coronary flow (i.e., Thrombolysis In Myocardial Infarction 0 and 1 flow) as compared to those with at least one SMuRF, while no independent associations were found between the presence or absence of SMuRFs and infarct size and left-ventricular ejection fraction after acute MI, assessed with cardiac magnetic resonance (CMR) [17]. An international study using CMR imaging confirmed the results of impaired pre-PCI coronary flow and similar left-ventricular damage in SMuRF-less patients [109]. Although a post hoc analysis of the DANAMI-3 trial showed that SMuRF-less patients had a larger infarct size and a smaller area of myocardial salvage on CMR following PCI, the association was mediated by a greater proportion of culprit vessel in the left anterior descending coronary artery and poor pre-PCI coronary blood flow [111]. Of note, patients with acute MI and no SMuRFs are predisposed to be presented with cardiogenic shock and cardiac arrest, leading to a poor prognosis in this patient subset. Although unestablished yet, we believe that the higher risks of cardiogenic shock and cardiac arrest may be attributed to no preconditioning in SMuRF-less patients and the potential underreporting of risk factors (Fig. 1). Acute MI can be driven by a sudden and aggressive thrombotic alteration with the lack of ischemic preconditioning in patients without SMuRFs [112]. In a previous study, the presence of pre-existing ischemic events and diseases was associated with better short-term prognosis after acute MI [113]. Another possible mechanism of severe clinical presentation in SMuRF-less patients with acute MI includes the underreporting of risk factors. It is conceivable that patients with severe clinical presentation of acute MI are likely to have incomplete medical records, particularly when they die shortly after admission [21, 114], potentially resulting in lower recorded rates of SMuRFs in patients with cardiogenic shock and cardiac arrest. Although the pathophysiology under acute MI without standard risks remains to be elucidated, comprehensive evaluation and scrutiny should be done when managing SMuRF-less patients and interpreting the results of studies focusing on no SMuRFs in acute MI [115].

### Prognosis of no SMuRFs after acute MI

The prognosis after acute MI in patients with no SMuRFs is counterintuitively worse than those with SMuRFs. Although some studies showed neutral results or even lower mortality rates in SMuRF-less patients [16, 19, 32], in-hospital or 30-day mortality after MI is higher when SMuRFs are absent (Table 1). Intriguingly, the prevalence of being SMuRF-less in acute MI may be lower, while the short-term mortality in patients with no SMuRFs might be higher in studies from Asian countries, especially in East Asia (Table 1). Overall, a meta-analysis demonstrated a 1.5-fold higher in-hospital mortality in SMuRF-less patients [85]. The sex difference in the prevalence of being SMuRF-less in patients with acute MI is unclear [85], and the impact of sex on short-term prognosis is complicated. The traditional understanding is that among general ACS and acute MI populations, women have a worse prognosis than men [116, 117]. Similarly, women without any SMuRFs reportedly have higher mortality after acute MI than men with no SMuRFs [15, 23, 28]. However, the “negative” prognostic impact of the lack of SMuRFs as compared to having such risk factors might be greater in men than in women [29]. Contrary to short-term mortality, the prognostic impact of being SMuRF-less on long-term outcomes after acute MI has not been established [22, 27]. The mechanisms of the higher in-hospital mortality are still unclear, but it may be associated with different patient profiles, severe clinical presentation, and less aggressive medical treatment in patients without SMuRFs. Acute MI in patients with no SMuRFs may be attributable to background cancer and CSIDs in up to one-third of cases [20], which are potentially associated with the high mortality rate. However, even after excluding patients with active cancer and CSIDs, the absence of SMuRFs was associated with increased in-hospital mortality in our previous report [106]. Another important point to note is that patients with no SMuRFs are less likely to receive intensive therapeutic strategies, including primary PCI and medications (e.g., angiotensin-converting-enzyme inhibitor, β-blocker, and statin) [19, 23, 85, 118]. Nonetheless, even when focusing only on patients undergoing PCI, the short-term prognosis after MI is worse in SMuRF-less patients [20, 24, 29, 31]. The lack of hypertension, diabetes, dyslipidemia, and current smoking as a therapeutic target presumably makes it challenging to improve clinical outcomes in SMuRF-less patients. Given that long-term outcomes do not differ considerably between patients with and without SMuRFs, it is currently uncertain whether the less frequent prescription of guideline-directed medical therapy in SMuRF-less patients has a prognostic impact, but to improve outcomes, guideline-recommended management should be considered in this vulnerable patient subset.

## Conclusions

Despite recent advances in the evidence-based management of acute MI, patients who develop coronary atherosclerosis and thrombotic events in the absence of traditional risk factors are underrecognized, and diagnostic and therapeutic pathways are unestablished in patients with MI and no SMuRFs. Although the prevalence, pathophysiology, and prognosis of SMuRF-less patients with acute MI have been increasingly reported, dedicated clinical studies are warranted to better define characteristics and outcomes and to clarify novel risk factors and specific secondary preventive strategies in this unique and vulnerable patient population.
